# Genome-wide linkage analysis combined with genome sequencing in large families with intracranial aneurysms

**DOI:** 10.1038/s41431-022-01059-0

**Published:** 2022-03-01

**Authors:** Mark K. Bakker, Suze Cobyte, Frederic A. M. Hennekam, Gabriel J. E. Rinkel, Jan H. Veldink, Ynte M. Ruigrok

**Affiliations:** 1 Department of Neurology, University Medical Center Utrecht Brain Center, Utrecht, the Netherlands; 2 Department of Genetics, Utrecht University Medical Center, Utrecht, the Netherlands

**Keywords:** Stroke, Aneurysm, Genetic linkage study, Next-generation sequencing

## Abstract

Rupture of an intracranial aneurysm (IA) leads to aneurysmal subarachnoid haemorrhage (ASAH), a severe type of stroke. Some rare variants that cause IA in families have been identified, but still, the majority of genetic causes, as well as the biological mechanisms of IA development and rupture, remain unknown. We aimed to identify rare, damaging variants for IA in three large Dutch families with multiple affected members with IA (*N* = 9, 11, and 6). By combining linkage analysis and genome sequencing (GS), we identified six rare and damaging variants for which all cases within one of the families were heterozygous. These variants were p.Tyr87Cys in *SYCP1*, p.Phe1077Leu in *FMNL2*, p.Thr754Lys in *TBC1D2*, p.Arg321His in *ZNF782*, p.Arg979Trp in *CCDC180*, and p.Val125Met in *NCBP1*. None of the variants showed association with IA status in a large cohort of 937 patients from the general IA patient population and 1046 controls. Gene expression in IA and cerebral artery tissue further prioritized *FMNL2* and *TBC1D2* as potential important players in IA pathophysiology. Further studies are needed to characterize the functional consequences of the identified variants and their role in the biological mechanisms of IA.

## Introduction

An intracranial aneurysm (IA) is a dilation of a cerebral blood vessel which are found in about 3% of the general population [1]. Rupture of an IA leads to aneurysmal subarachnoid haemorrhage (ASAH), a severe type of stroke with an incidence of 6.1 per 100,000 person-years [2]. ASAH is a life-threatening event with substantial morbidity and mortality with one in three patients dying [3]. Of the patients who survive ASAH, approximately half remain dependent on help from others in daily life [3]. Endovascular or surgical treatment of IA can prevent ASAH, thus avoiding its devastating consequences. However, currently, no clinically relevant risk models are available to identify persons at risk of developing and rupturing of IA.

The most important risk factors for IA and ASAH are age, female sex, hypertension, smoking, and a positive family history for ASAH [1, 2]. In persons with two or more first-degree family members with ASAH the life-time risk of ASAH is 50 times higher than in persons without such a family history [1, 4]. Moreover, individuals with an IA and a positive family history for ASAH have an almost 3-fold increased risk of rupture compared to individuals with IAs without such a family history (i.e., sporadic IAs) [5]. The heritability of ASAH has been estimated at 41% [6] and genome-wide association studies (GWAS) of large cohorts of IA cases have identified many common genetic risk factors with small effects, which combined explain roughly half of the total heritability [7]. Besides GWAS, sequencing studies in families with multiple affected family members with IA have identified rare IA-causing genetic variants with a presumable large effect located in the following genes: *ADAMTS15* [8], *THSD1* [9], *ANGTL6* [10], *PCNT* [11], and *ARHGEF17* [12]. For the genes *THSD1* and *ADAMTS15*, linkage analyses had previously already highlighted regions containing these genes [13, 14], showing that linkage analysis with subsequent sequencing is a viable method to identify risk genes for IA.

Despite recent findings of IA risk genes and their associated biological functions, the primary causes of IA remain unknown. Here, we aim to identify rare, damaging genetic variants segregating with IA in three large Dutch families with multiple affected first-degree family members with an unruptured IA or ASAH using a combination of linkage analysis and genome sequencing.

## Methods

**Fig. 1:**
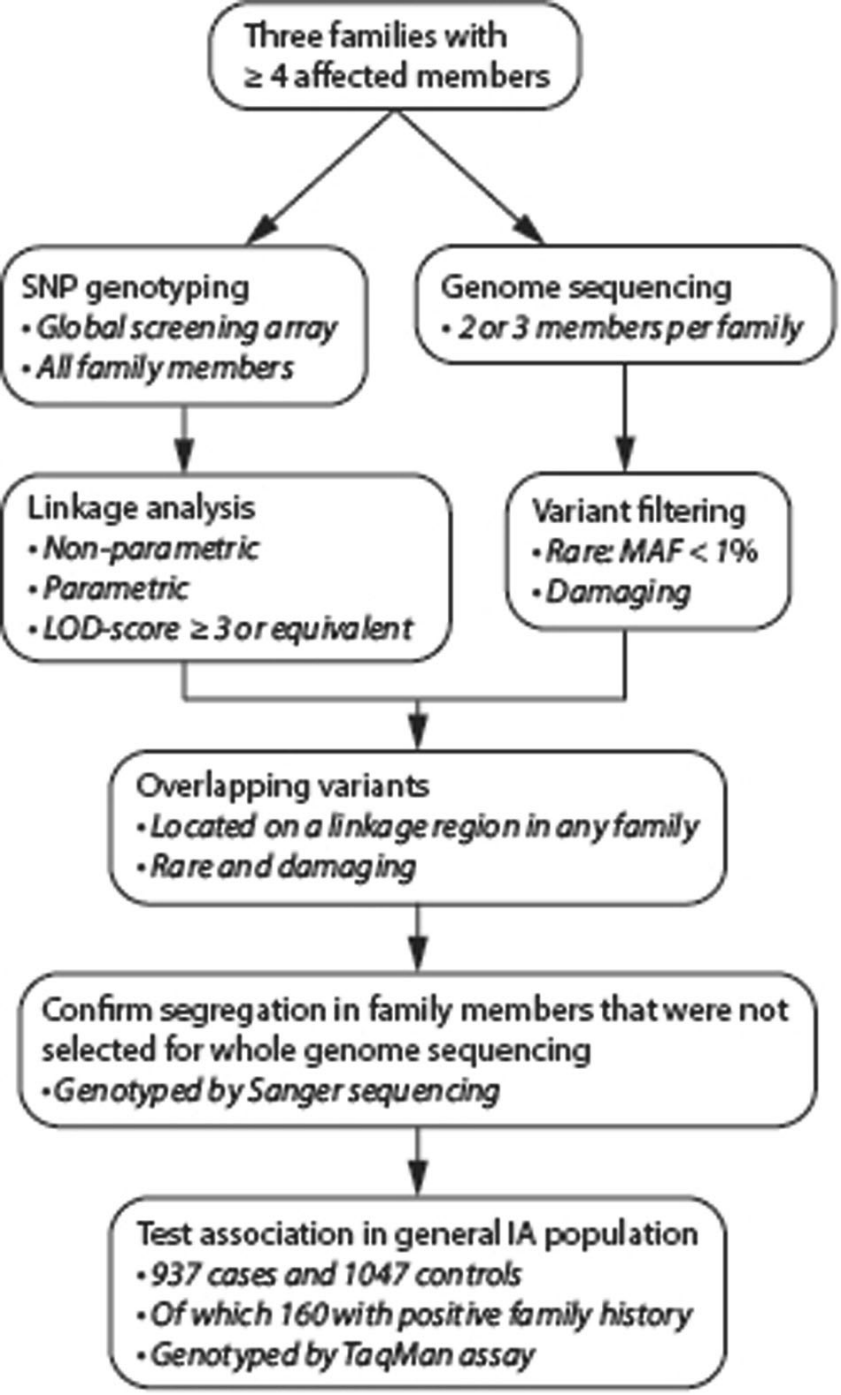
Flowchart of the methods in this study. *SNP* Single-nucleotide polymorphism. *MAF* minor allele frequency. *LOD* logarithm-of-odds.

A flowchart of the methods is shown in Fig. 1.

### Participants

**Fig. 2:**
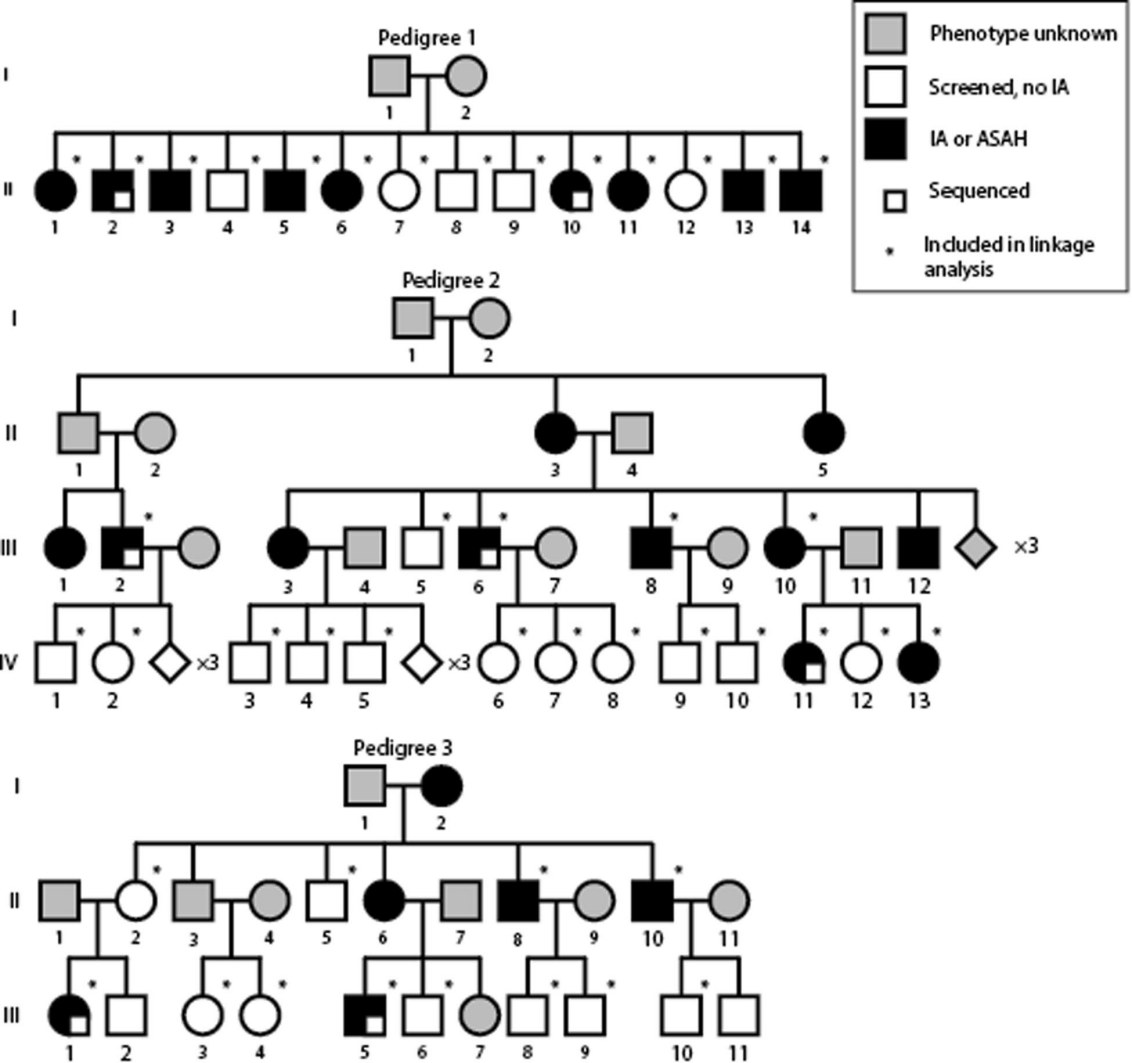
Families recruited for linkage analysis and GS. Black: affected with an unruptured IA and/or ASAH. Grey: not included. White: no IA found at time of screening. Small white square: included in GS. Persons included in linkage analysis are depicted by an asterisk.

Three large Dutch families with multiple affected first-degree relatives with an unruptured IA or an ASAH were selected: pedigree 1 included nine affected relatives in one generation, pedigree 2 included eleven relatives in three successive generations, while pedigree 3 included six in three successive generations (Fig. 2). Genealogical research showed no shared ancestor between these families for at least six generations back. For the variants identified in these families we further determined their role in the general IA patient population, using a cohort of 937 Dutch IA patients, of whom 160 had a positive family history for ASAH, from the University Medical Center Utrecht (Table 1). For the control cohort, GS genotypes from 1046 persons recruited within Project MinE were used [15, 16]. Presence of IA was determined with computed tomography, magnetic resonance, or digital subtraction angiogram. Families or persons with Loeys-Dietz syndrome, Ehlers-Danlos disease, Marfan Syndrome, or autosomal dominant polycystic kidney disease were excluded.

**Table 1:** General IA patient cohort characteristics.

Characteristic	Value
Participants (*N*)	937
Women. *N* (%)	663 (70.8%)
ASAH. *N* (%)	644 (69.3%)
Age at ASAH in years [mean (standard deviation)]*	56.6 (13.4)
Positive family history. *N* (%)	160 (18.1%)
Hypertension	376 (40.7%)
Ever smoked	641 (71.6%)
Number of IAs. *N* (%)	
1	707 (76.0%)
2	160 (17.2%)
3	47 (5.1%)
≥4	16 (1.7%)

ASAH: aneurysmal subarachnoid haemorrhage. Percentage multiplied by value may not add up to the total *N* due to missing values. *Available for 643 patients with ASAH.

### Linkage analysis

The following number of family members were included for genotyping: nine affecteds and five unaffecteds from pedigree 1; six affecteds and 12 unaffecteds from pedigree 2; and four affecteds and eight unaffected from pedigree 3 (Fig. 2). DNA was extracted from blood with the Chemagic DNA Blood10k kit (PerkinElmer, Inc., Waltham, MA, USA). Single nucleotide polymorphism (SNP) genotyping was performed with the Global Screening Array (Illumina, San Diego, CA, USA). Detailed methods on the SNP quality control are described in the Supplementary Note. Two linkage methods were performed. First, we performed non-parametric linkage (NPL) analysis as it is an effective method to detect segments of DNA that are identical-by-descent between affected family members. Due to the age-dependent penetrance of IA, unaffected family members are may also be heterozygous for a pathogenic variant, making the use of unaffected family members in NPL analysis ineffective. Therefore, the second method, parametric linkage analysis, was used to specify disease penetrance values depending on age categories and/or other variables, thereby allowing the inclusion of unaffected family members.

Non-parametric linkage analysis was performed using MORGAN *lm_ibdtest* [17]. MORGAN uses a Markov Chain Monte Carlo approach to estimate inheritance vectors: a representation of allele sharing by descent. In this iterative process, the first 5,000 iterations were discarded, before saving every 30^th^ iteration from a total of 90,000 iterations for analysis. NPL scores Spairs and Srobdom were calculated [18, 19]. Spairs can detect both recessive and dominant inheritance loci, while Srobdom has the most power to detect linkage in a dominant model [19].

Parametric linkage analysis was performed in three steps: (1) obtaining inheritance vectors using MORGAN *gl_auto*, (2) determining pedigree baseline log likelihoods with MORGAN *base_trait_lods*, and (3) calculating logarithm of odds (LOD) scores with MORGAN *gl_lods* [20]. The *gl_auto* option was run with the same setting to estimate inheritance vectors as non-parametric linkage analysis. Four inheritance models; two dominant inheritance models and two recessive models, with either low or high penetrance, were used to calculate LOD-scores, using the liability class-specific penetrance matrix option of MORGAN. Phenocopy rate was set at the population prevalence of 3% [1]. Prevalence of IA tends to increase with age until, but the precise interaction between age and penetrance of high impact genetic variants is unknown [1]. Therefore, we selected two age-dependent penetrance models, reaching a maximum penetrance at age 70 (95% for the high penetrance models, 80% for the low penetrance models), and decreasing by 15% each younger decade until reaching the phenocopy rate (Supplementary Note). Expected allele frequency of the trait locus was set at 0.1% for dominant models and 1% for recessive models.

Regions with statistically significant linkage were defined as having LOD ≥ 3.3, or an equivalent NPL-score ≥ 3.90, derived from [21]:

$$LOD = \frac{{NPL^2}}{{2 \times \ln 10}}$$

Or equivalent:

$$NPL = \sqrt {LOD \times 2 \times \ln 10}$$

Regions with suggestive statistically significant linkage were defined as LOD ≥ 1 or NPL-score ≥ 2.15.

### Genome sequencing

From each family, two or three of the affected family members were selected for genome sequencing (GS), totaling seven patients (Fig. 2). Library preparation and sequencing was performed by Macrogen Inc. (Seoul, South Korea). The TruSeq DNA PCR-Free Shotgun Library was used on 350 bp fragments. Sequencing was done on the Illumina HiSeq system. Detailed methods about mapping, variants calling and quality control of GS data are given in the Supplementary Note.

### Variant selection

After variant calling, variants located on regions identified by linkage analysis with suggestive statistical significance (LOD ≥ 1 or NPL-score ≥ 2.15) and for which all affected members of the family in which the region was identified were heterozygous, were retained. We also included variants located on previously identified linkage loci (loci from ref. [22]. and specified in Supplementary Table 1). SnpEff was used to annotate variants (more details in the Supplementary Note) [23]. Variants in loci with suggestive significance were selected if they (1) had MAF ≤ 1% in non-Finnish Europeans in ExAC [24], Dutch controls from Project MinE [16], and Dutch controls from GoNL [25], or were not reported in any of these datasets, and (2) were disruptive of non-disruptive missense, which included any of the following SnpEff annotations: exon loss, frameshift, splice acceptor, splice donor, start site loss, stop site gain, stop site loss or transcript ablation, rare amino acid variant, inframe deletion, inframe insertion, or missense.

### Sanger sequencing

In order to validate variants found using GS, and to test segregation in additional affected and unaffected family members, we used Sanger sequencing. In pedigree 1 an additional seven affected family members (resulting in nine affected family members with genotyping information in total) and five unaffected members were included; in pedigree 2 an additional three affected (six in total) and twelve unaffected, and in pedigree 3 an additional two affected (four in total) and eight unaffected members were included. PCR primers were designed using Primer3Plus, or if not possible by manual selection. Surrounding sequences (500 bp on both ends) were obtained from Ensembl v99. In order to design primers and probes that only bind to positions without genetic variants, the genetic variants with MAF ≥ 0.01% were converted to an *N* before uploading in the TaqMan assay design tool. An overview of the primers is shown in Supplementary Table 2. Primer annealing temperature was optimized for each primer pair, by selecting the temperature resulting in the strongest specific amplification, as determined by gel electrophoresis. Genotypes were called by visually inspecting the Sanger sequencing chromatograms.

### TaqMan assay

Segregating variants identified by linkage analysis and GS and validated using Sanger sequencing were additionally genotyped in a large cohort of 937 IA patients (Table 1) using TaqMan assay (Thermo Fisher Scientific Inc. Waltham, MA, USA). Details on assay design and optimization are given in the Supplementary Note. Quantitative polymerase chain reaction was conducted using the QuantStudio 6 Flex Real-Time PCR system (Applied biosystems). Polymerase chain reaction protocol was as follows: 10 min at 95 °C, 40 cycles of [15 s at 92 °C, 1 min at 60 °C], and 1 min at 60 °C. Fluorescence was read by the QuantStudio 6 Flex Real-Time PCR system and analyzed using the QuantStudio 6 and 7 system software. Genotypes of Dutch controls from Project MinE were obtained by GS as described before [15]. Association of the variants with IA was determined using two-tailed Firth logistic regression in PLINK v2.0. Sex and the first 10 genetic principal components projected on the HapMap v3 reference dataset were used as covariates. Only European ancestry individuals were included. These were defined by a maximum of five standard deviations distance from the mean of the genetically Dutch control samples from Project MinE in principal components 1–4. Since only persons homozygous for the ancestral allele, or heterozygous, were observed, a dominant inheritance model was selected. The analysis was also performed including only cases with positive family history versus controls. A *P*-value of 0.05 divided by the number of genes included with at least one observed mutated allele (*N* = 5) was considered statistically significant.

### Rare variant association and burden analysis

To assess if additional rare variants in the genes containing potentially pathogenic variants segregating with IA, may be associated with IA, we performed two analyses using exome sequencing data from the UK Biobank: a rare variant burden analysis and a variant-level association analysis of potentially pathogenic variants. Variants were annotated based on predicted functional impact using SNPEff [23]. The association between IA and the number of rare, coding, minor alleles of the selected genes was calculated by two-tailed Firth logistic regression. For the variant-level association analysis, variants predicted to have a high functional impact, and a minor allele frequency below 1% were selected. Association was assessed using two-tailed Firth logistic regression. Statistical significance for the variant-level association analysis was defined as a *P*-value below 0.05 divided by the number of variants tested for that gene. More details on both analyses are given in the Supplementary Note.

### Gene expression analysis

For the genes in which segregating variants were found, we performed a lookup of the gene expression, and of differentially expressed genes between IA tissue and control cerebral artery tissue. We used RNA sequencing read counts, and differential expression data for IA tissue and control cerebral artery tissue analyzed and described before [26]. Differential expression with a false discovery rate below 0.05 was considered statistically significant.

## Results

### Linkage analysis

After genotype quality control 162,641 SNPs and all 19 cases and 25 controls remained for linkage analysis. Non-parametric linkage analysis identified 12 regions with NPL-score ≥ 3.90 (equivalent to LOD ≥ 3.3; statistically significant linkage) and 51 regions with NPL-score ≥ 2.15 (LOD ≥ 1.0; suggestive statistically significant linkage) across the three families (Table 2 and Supplementary Table 3 for genome-wide NPL scores and full linkage statistics). All statistically significant linkage regions were identified using a dominant inheritance model (the Srobdom statistic), while one of these regions was also detected using a recessive model (the Spairs statistic). Parametric linkage analysis also identified five of these regions with at least suggestive evidence for linkage (2q23.3–24.1 and 2q35 in pedigree 1, 10q22.1–22.2 and 14q12–13.1 in pedigree 2, and 5q14.2–14.3 in pedigree 3), but did not identify any additional regions of interest (Supplementary Table 4). A total of 39 regions with suggestive statistical significance (LOD ≥ 1.0) were found using parametric linkage analysis.

**Table 2:** Linkage analysis regions with LOD-score ≥ 3.3.

Pedigree	Chromosome	Position	Locus	Statistic	Highest NPL	Previous evidence
1	2	150020329– 159204593	2q23.3–24.1	Srobdom	7.34	Hypertension linkage analyses. IA GWAS
	2	217211217– 218756117	2q35	Spairs and Srobdom	7.48	
	9	93471627– 101092018	9q22.2–22.33	Srobdom	7.24	Thoracid aortic aneurysm/Loeys Dietz syndrome, coronary artery disease, IA.
2	9	24756027– 25973292	9p21.3–21.2	Srobdom	6.00	Near, but > 2 megabases away, the 9p GWAS locus associated with many cardiac traits
	10	72515775– 77216955	10q22.1–22.2	Srobdom	6.92	Thoracic aortic aneurysm (*ACTA2*)
	14	28788618– 34682723	14q12–13.1	Srobdom	6.73	
3	5	81207272– 90645137	5q14.2–14.3	Srobdom	5.15	IA (Exome sequencing study, *EDIL3*)
	5	171960073– 174241185	5q35.1–35.2	Srobdom	5.21	
	6	84533341– 94104527	6q14.2–16.1	Srobdom	4.40	3 megabases away from IA GWAS locus
	7	36588659– 39942647	7p14.2–14.1	Srobdom	5.26	
	20	53425217– 54482619	20q13.2	Srobdom	5.28	
	22	48258293– 51151350	22q13.31-13.33	Srobdom	5.37	

Regions are defined as least 3 consecutive markers passing this threshold, with gaps allowed if all markers in between have LOD > 1. Positions on GRCh37. Previous evidence are prior associations with IA, another aneurysm type, or another cerebrovascular disease.

### Rare variant discovery by genome sequencing

Eleven variants on four distinct regions with at least suggestive evidence for linkage (Supplementary Tables 3 and 4) passed the selection criteria for potential disease-causing variants in any of the families: one variant on locus 1p13.1–31.1 identified in pedigree 3, two on 2q23.3–24.1 identified in pedigree 1, one on 8p22.2 (and previously identified by Kim et al. [27]) and found in pedigree 1, and seven on 9q22.2–22.33 identified in pedigree 1 (Supplementary Table 5). On region 9q22.2–22.33, variant *COL15A1* p.Lys1001Arg was found in all affected members of pedigree 2, while the region was identified by linkage in pedigree 1. Sanger sequencing in the remaining family members confirmed segregation of six of the 11 variants among all affected members, while for the other five, not all cases among one family were heterozygous, and these variants were excluded from further analysis (See Table 3 for both segregating and not segregating variants). The six variants that remained were: *SYCP1* p.Tyr87Cys on 1p13.1–31.1 in pedigree 3, *FMNL2* p.Phe1077Leu on 2q23.3–24.1 in pedigree 1, and four variants on 9q22.2–22.33 in pedigree 1: *TBC1D2* p.Thr754Lys, *ZNF782* p.Arg321His, *CCDC180* p.Arg979Trp, and *NCBP1* p.Val125Met. All affected family members in pedigree 1 were heterozygous for both *FMNL* p.Phe1077Leu and *TBC1D2* p.thr754Lys. Among the unaffected members of pedigree 1, one was heterozygous for both variants. Despite this genotype, this male member (II-8) had a negative screen for IA at age 65. Three unaffected family members were not heterozygous for either variant (II-4, II-7, and II-12), and one for neither (II-9).

**Table 3:** Results of the segregation analysis of variants identified by genome sequencing, in additional family members.

Segregating variant	Pedigree	variant ID	Transcript variant	Protein variant	Max. NPL	MAF	Gene	Heterozygous cases	Heterozygous controls
Yes	3	1:114857466:A:G	NM_003176.3:c.260A > G	NP_003167.2:p.(Tyr87Cys)	3.23 (Srobdom)	0.0043	*SYCP1*	4/4	1/8
	1	2:152647857:T:G	NM_052905.3:c.3231T > G	NP_443137.2:p.(Phe1077Leu)	7.34 (Srobdom)	0.0028	*FMNL2*	9/9	2/5
	1	9:96819061:C:T	NM_001001662.1:c.962G > A	NP_001001662.1:p.(Arg321His)	7.24 (Srobdom)	0.0071	*ZNF782*	9/9	3/5
	1	9:97350488:C:T	NM_020893.2:c.3067C > T	NP_065944.2:p.(Arg1023Trp)	7.24 (Srobdom)	0.0018	*CCDC180*	9/9	3/5
	1	9:97643352:G:A	NM_002486.4:c.373G > A	NP_002477.1:p.(Val125Met)	7.24 (Srobdom)	0.0022	*NCBP1*	9/9	3/5
	1	9:98203298:G:T	NM_001267571.1:c.2261C > A	NP_001254500.1:p.(Thr754Lys)	7.24 (Srobdom)	–	*TBC1D2*	9/9	3/5
No	1	2:165933044:C:T	NM_024753.4:c.724G > A	NP_079029.3:p.(Asp242Asn)	7.34 (Srobdom)	0.0069	*TTC21B*	8/9	3/5
	1	8:13099452:C:A	NM_182643.2:c.2885G > T	NP_872584.2:p.(Ser962Ile)	3.61*	1.50 x 10^−5^	*DLC1*	7/9	4/5
	1	9:92616050:G:C	NM_022755.5:c.1258C > G	NP_073592.1:p.(Gln420Glu)	7.24 (Srobdom)	0.0033	*IPPK*	5/9	2/5
	1	9:95447208:G:A	NM_001354918.1:c.3892C > T	NP_001341847.1:p.(Arg1298Trp)	7.24 (Srobdom)	0.0014	*PTCH1*	5/9	3/5
	2	9:99054627:A:G	NM_001855.4:c.3002A > G	NP_001846.3:p.(Lys1001Arg)	7.24 (Srobdom)**	0.0095	*COL15A1*	5/6	4/12

All variants that are tested for segregation by Sanger sequencing are shown. Segregation is defined as all cases being heterozygous for the risk allele within the respective pedigree. Variant ID position on GRCh38. Max. *NPL* maximum non-parametric linkage score of the linkage region that identified the locus and for the linkage statistic this was found. *MAF* minor allele frequency in non-Finnish European persons included in the Exome Aggregation Consortium (ExAC). *Identified by Kim et al. Two-point logarithm of odds. **Region of linkage was identified in pedigree 1.

### Association of segregating variants in the general IA patient population

In the cohort of 937 IA patients (including 777 sporadic and 160 familial patients), for all variants except *TBC1D2* p.Thr754Lys, at least one person was heterozygous for the risk allele among both cases and controls (Table 4), but none of the variants showed a statistically significant association with IA. When analysing only cases with a positive family history for ASAH also no association was found. In the UK Biobank dataset, no statistically significant rare variant burden was found for gene containing one of the segregating variants (Supplementary Table 6), and no additional potentially pathogenic variants with a statistically significant association with IA status were found (Supplementary Table 7).

**Table 4:** Variant associations in the general IA patient cohort.

Gene	variant ID	Carriers among controls (*N* = 1047)	Carriers among cases (*N* = 937)	Carriers among familial cases (*N* = 160)	All IA		Familial IA	
					OR (95%CI)	*P*	OR (95%CI)	*P*
*TBC1D2*	9:98203298:G:T	–	0	0	–	–	–	–
*ZNF782*	9:96819061:C:T	16	9	2	1.03 (0.43– 2.54)	0.95	1.11 (0.26– 4.84)	0.89
*CCDC180*	9:97350488:C:T	8	5	1	0.59 (0.16– 2.16)	0.43	0.71 (0.10– 4.97)	0.73
*NCBP1*	9:97643352:G:A	11	5	1	0.81 (0.27– 2.43)	0.71	1.08 (0.17– 6.77)	0.94
*FMNL2*	2:152647857:T:G	16	8	1	0.68 (0.28– 1.68)	0.41	0.47 (0.077– 2.83)	0.41
*SYCP1*	1:114857466:A:G	13	4	0	0.27 (0.08– 0.94)	0.04	0.13 (0.01–2.46)	0.17

Positions on GRCh38. *IA* intracranial aneurysm. *OR* odds ratio from Firth logistic regression. 95%CI: 95% confidence interval.

### Gene expression in intracranial aneurysm and cerebral artery tissue

The genes *FMNL2* and *TBC1D2* had high median expression levels (among top 20% highest expressed genes with non-zero median expression) in IA tissue as well as control cerebral artery tissue (median RPKM = 11.1 [range = 1.18–38.5] and 10.3 [1.55–61.9], respectively in the combined tissues), whereas *NCBP1* and *ZNF782* showed a lower expression and *SYCP1* was expressed in too low amounts to be reliably detected (Supplementary Table 8). Differential expression analysis of these genes did not show difference in expression between aneurysms and control tissue for any of the genes.

## Discussion

Using genome-wide linkage analysis combined with GS in large families with multiple affected members with IA, we identified six rare, damaging variants located in the genes *SYCP1*, *FMNL2*, *TBC1D2*, *ZNF782*, *CCDC180*, and *NCBP1* which segregated among all cases of one of these families. All genes except *SYCP1* were expressed in intracranial aneurysm and control cerebral artery tissue, while *FMNL2* and *TBC1D2* showed high gene expression in these tissues, further prioritizing these genes. Genotyping the identified variants in a large cohort of IA patients did not show additional evidence that these variants play a role in IA patients beyond the families in which they were discovered.

Of the rare, damaging variants we identified, five were located on either of two regions with strong evidence (2q23.3–24.1 and 9q22.2–22.33), and one on a region with suggestive evidence (1p13.1–31.1). By Sanger sequencing we confirmed that all affected family members of the respective families were heterozygous for the identified variants. For *SYCP1* p.Tyr87Cys on 1p13.1–31.1, all four affected family members of pedigree 3 and only one of eight unaffected members of this family was heterozygous for the mutated allele. This is a reasonably strong segregation, but the number of affected members included remains too low to consider the genetic cause in this family resolved. For pedigree 1, all affected family members were heterozygous for variants on two linkage loci, 2q23.3–24.1 and 9q22.2–22.33. This means that all affected family members were heterozygous for both haplotypes. Whether this means that a genetic variant on both loci is required to develop and IA, or whether one of the loci is a false positive cannot be determined.

Variant *FMNL2* p.Phe1077Leu is located in the diaphanous autoregulatory domain of Formin-like protein 2. The protein interacts with Rho-GTPases and plays a role in nonbranched actin filament elongation and stability, especially in lamellipodia and filopodia [28–30]. Similar to IA-associated Rho-GTPase *ARHGEF17*, of which pathogenic variants cause intracranial haemorrhage in zebrafish, this could indicate that IA formation or rupture is mediated through actin-dependent smooth muscle cell action [10, 12]. The protein product of *TBC1D2* also influences GTPase activity, through activation of the GTPase of Ras-related protein Rab-7a, and thereby reducing cell-cell adhesion. Furthermore, it is involved in cadherin-dependent cell-cell adhesion [28, 31]. Variant *TBC1D2* p.Thr754Lys is located in the Rab-GTPase-TBC domain. In the rat, genetic variation in a large region surrounding ortholog Tbc1d2 was strongly associated with ruptures of the internal elastic lamina of the aorta and iliac arteries [32]. The role of this gene is largely unclear, but could indicate that it provides IA risk through impaired elastic lamina integrity. Variant *NCBP1* p.Val125Met is located in nuclear cap-binding protein subunit 1 gene in the domain ‘middle domain of eukaryotic initiation factor 4G’ (MIF4G). The protein product is part of the mRNA cap-binding complex and involved in a range of mRNA processes [28, 33]. It is unclear how this gene might contribute to IA pathophysiology. The protein products of *SYCP1* and *ZNF782* are involved in DNA binding, without apparent implications in blood vessel or aneurysm biology [28]. The role of *CCDC180*, also known as *BDAG1*, is not well understood.

Several of the regions identified by linkage analysis in our study were previously already implicated to play a role in IA: some were identified in former genetic analyses of IA and some in genetic analyses of related diseases. The 9q22.2 locus identified in pedigree 1 maps to a locus identified by a GWAS of IA in the Korean population [34]. The locus was further linked to coronary artery disease by linkage analyses in 24 large and in 428 multiplex families (Two-point NPL-scores of 4.54 and 3.72, respectively) [35, 36]. The locus contains *TGFBR2*, a gene of which pathogenic variants are the cause for 25% of cases of Loeys Dietz syndrome, which predisposes mainly to thoracic aortic aneurysms but also to IA [37–39]. Here, no potential disease-causing variants in *TGFBR2* were found. Instead, we found four rare, damaging variants on this locus that segregated with disease status in pedigree 1: *TBC1D2* p.Thr754Lys, *ZNF782* p.Arg321His, *CCDC180* p.Arg979Trp, and *NCBP1* p.Val125Met. In the same family, linkage was also found on locus 2q23.3–24.1. This locus was associated with IA in a GWAS of a Finnish population isolate [40] and has been linked to chronic hypertension [41–43]. Another locus identified in this study, 10q22.1–22.2, contains the *ACTA2* gene, of which pathogenic variants are the cause 14% of familial thoracic aortic aneurysms and dissection [44]. Here, we were not able to identify rare, damaging variants in *ACTA2* or any other gene in this locus. The 5q14.2–14.3 locus contains the *EDIL3* gene. An exome sequencing study on a family of three members with ASAH found that *EDIL3* p.Cys128Tyr was shared by all cases. In a cohort of 35 unrelated IA cases, the researchers found another variant in *EDIL3* (p.Lys387Gln) in 2 cases [45]. Here, we did not find any rare, damaging variants in *EDIL3*, nor in other genes in this locus. Locus 9p21.3 is a well-known GWAS risk locus for IA and several other (cerebro-)vascular diseases [7, 46, 47], and locus 6q16.1 was also been identified as risk variant in GWAS of IA [7]. Both regions identified in this study are located ± 3 megabases away from the lead GWAS variants, which could mean that these are located too far away to be driven by the same causal variants as the GWAS loci. No rare, damaging variants were found in these regions.

This study has considerable strengths. First, the combination of linkage analysis with genome sequencing in large families provides an opportunity to detect rare damaging variants on segregating genomic regions. Second, using GS instead of exome sequencing provides a more uniform coverage of the genome and can detect more coding variants than exome sequencing [48], which was used in previous IA sequencing studies [8–12]. Another strength is the use of MORGAN for the linkage analysis. This allows joint analysis of the full pedigrees and leverages dense SNP information to provide high-resolution allele sharing information.

Some limitations apply to this study. First, the genetic association between variants discovered in this study and IA does not prove a causal relationship. Functional follow-up studies are needed to further prioritize these variants. Second, most regions of linkage were identified through an NPL statistic optimized for dominant inheritance (Srobdom), while only one and five of those were also identified using the Spairs statistic or parametric linkage analysis, respectively. Srobdom uses an exponential function that increases quickly with the number of affected persons who share an allele identical-by-descent [19]. This can lead to very high Srobdom in large pedigrees with many closely related persons such as pedigree 1 in this study. To deal with this, we used parametric and non-parametric linkage analysis, allowing for both dominant and recessive inheritance (by defining penetrance categories for parametric linkage analysis, and Spairs and Srobdom for NPL) for the purpose of detecting regions of linkage, and subsequently validated segregation among all affected members of a family using Sanger sequencing. Thereby we identified regions truly shared identical-by-descent among all affected family members, and thus eliminating this potential limitation. Another limitation is that we identified multiple variants of interest within a single locus of linkage, in particular 9q22.2–22.33. It can be expected that only a single causal variant is present on this locus, indicating the majority of variants segregates with IA simply by being in close proximity to this variant and are not causal. Including additional family members to increase resolution, identifying structural genetic variants, and performing functional experiments are ways to further prioritize variants in this locus.

In summary, we conducted a genome-wide linkage analysis paired with GS in three large families with IA and ASAH patients. This led to the identification of six potential IA-causing variants in families. We did not find evidence that these variants also contribute to IA in the general IA population. Regions of linkage showed overlap with previous genetic studies of IA and cerebrovascular and connective tissue disorders, providing insight in the pathogenic mechanisms of IA. As a future direction of research, we suggest functional follow-up studies to discover the roles of the IA-associated genes, in particular *FMNL2* and *TBC1D2* which are highly expressed in cerebral artery and intracranial aneurysm tissue, and to discover how the damaging variant in those genes contribute to IA pathophysiology.

## Supplementary information

Supplementary Note

Supplementary Table 3

Supplementary Table 4

Supplementary Table 7

## Data Availability

The datasets generated and/or analyzed during the current study are available from the corresponding author on reasonable request.
