# Supplementary material for: Genome-wide linkage analysis combined with genome sequencing in large families with intracranial aneurysms
Source: Eur J Hum Genet. 2022 Mar 1;30(7):833–40. doi: 10.1038/s41431-022-01059-0 (PMC9259640; doi:10.1038/s41431-022-01059-0)
Supplement: Supplementary file 1 — Supplementary Note [file 41431_2022_1059_MOESM1_ESM.docx]

Supplementary Data

Genome-wide linkage analysis combined with genome sequencing in large families with intracranial aneurysms

# Authors and affiliations

Mark K. Bakker^1^, Suze Cobyte^1^, Frederic A. M. Hennekam^2^, Gabriel J.E. Rinkel^1^, Jan H. Veldink^1*^, Ynte M. Ruigrok^1*^

**^1^** Department of Neurology, University Medical Center Utrecht Brain Center, Utrecht, the Netherlands.

**^2^** Department of Genetics, Utrecht University Medical Center, Utrecht, the Netherlands.

*These authors jointly supervised this work.

Table of Contents

[Authors and affiliations 1](#_Toc84333756)

[Supplementary Methods 2](#_Toc84333757)

[Single nucleotide polymorphism quality control 2](#_Toc84333758)

[SNP selection for linkage analysis 3](#_Toc84333759)

[Linkage analysis penetrance categories 3](#_Toc84333760)

[Genome sequencing data mapping 3](#_Toc84333761)

[WGS variant calling 3](#_Toc84333762)

[WGS quality control 4](#_Toc84333763)

[TaqMan assay design and optimization 4](#_Toc84333764)

[Supplementary Figures 6](#_Toc84333765)

[Supplementary Tables 12](#_Toc84333766)

[References 15](#_Toc84333767)

# Supplementary Methods

## Single nucleotide polymorphism quality control

The samples were genotyped as part of a larger batch of 1025 samples. The following SNP genotype quality control steps were performed on the entire set. Samples with missingness > 4% or single nucleotide polymorphisms (SNPs) with missingness > 7.5% were removed using plink v1.90. A sexcheck and relatedness test was performed and potential sample mixups and duplicates were excluded. Using a pruned set of SNPs (*plink --indep-pairwise 50 5 0.5*), relatedness and heterozygosity were determined. Samples with a heterozygosity F-statistic < -0.025 or F > 0.05 were removed. Relatedness between family members was confirmed, and no additional family members were identified. Ancestry was determined by calculating genetic principal components projected on samples from the HapMap project v3. Individuals more than 10 standard deviations away from the mean of the European HapMap samples on principal components 1-4 were excluded. Next, SNPs with a minor allele count below 10, biased missingness false discovery rate below 0.001 or Hardy-Weinberg disequilibrium P-value below 1^.^10^-10^ were excluded.

The following SNP genotype quality control steps were only performed in samples from the three IA families. Genotyping errors that violate Mendelian inheritance were identified. Mendelian inconsistencies (i.e. genotypes that directly violate Mendelian inheritance) were detected and set to missing using *plink --mendel-duos --mendel-multigen --set-me-missing* flags. Mendelian consistent errors (where Mendelian inheritance violation can only be detected from inferred haplotypes) were detected using GIGI-check 1.06^1^ and MORGAN v3.3.^2^ MORGAN’s *gl_auto* uses Markov Chain Monte Carlo to determine likelihoods for inheritance vectors. Since GIGI-check is based on the assumption of no genotyping error, which is usually not true for genotyping data, we used three distinct genotype frameworks. Each of SNPs on average 2 cM apart with a minor allele frequency (MAF) above 10% in all global screening array samples. A burn-in of 1,000 iterations and analysis of 30,000 iterations was used, storing output of every 30th iteration. SNPs with a probability of no error below 0.05 in at least two frameworks were excluded.

## SNP selection for linkage analysis

SNPs that passed genotyping quality control as outlined in the previous paragraph were pruned using *plink --indep-pairwise 50 5 0.1*. SNPs with MAF below 0.05 in the Global Screening Array reference set were excluded. Sex-specific Kosambi positions (<http://compgen.rutgers.edu/download_maps.shtml>) were interpolated for all genotyped variants using R.

## Linkage analysis penetrance categories

Penetrance category was based on age decade bins: for the low penetrance models, penetrance was 3% (phenocopy rate) until age 20, 5% for ages 20-30, 20% for ages 30-40, 35% for ages 40-50, 50% for ages 50-60, 65% for ages 60-70, and 80% for ages 70 and up, and for the high penetrance models this was 3% until age 10, 5% for ages 10-20, 20% for ages 20-30, 35% for ages 30-40, 50% for ages 40-50, 65% for ages 50-60, 80% for ages 60-70, and 95% for ages 70 and up.

## Genome sequencing data mapping

Genome sequencing resulting in two paired fastq files per sample used to mapping. Quality of fastq files was assessed using fastqc.^3^ The samples were mapped to genome build GRCh38 using a lossless genomic analysis toolkit version 4 (GATK4)^4^ best practices workflow for germline variant detection.^5^ Fastq files were converted to Bam with Picard 2.17.10 FastqToSam.^6^ Illumina adapters were marked with Picard MarkIlluminaAdapters. Adapters were clipped with Picard SamToFastq, aligned with BWA-MEM 0.7.12, and a bam file containing all original reads was created with Picard MergeBamAlignment. A final unmapped bam file was made with Picard RevertSam.

## WGS variant calling

Genetic variants were called per sample according to GATK Best Practices. Unmapped bam files were aligned to GRCh38 with BWA-MEM. Duplicate reads and their unmapped pair mates were flagged using picard MarkDuplicates. Base quality score recalibration was done using GATK v4.0.20 ApplyBQSR. SNPs, insertions and deletions were called using the HaplotypeCaller option from GATK v3.5.0. Then, the GATK Best Practices pipeline for joint variant discovery was used to combine gvcf files and assess per variant quality scores. GVCF files were joined using GATK v4.0.20 GenotypeGVCFs. A VCF file with only variants was created using GATK v4.0.20 MakeSitesOnlyVcf. Then, GATK v4.0.20 VariantRecalibrator was called twice to assess variant quality scores for SNPs, and indels.

## WGS quality control

Variant effect information was annotated using SnpEff 4.3.^7^ Only predictions on protein coding canonical transcripts (build GRCh38.86) were maintained. Variants with a SnpEff warning or error, multi-allelic variants, variants with missing data for more than 2 samples (13.3%), variant quality score below 30, or genotype quality score above 10 were excluded.

A high-quality truth set was used assess sample quality. Hapmap v3 SNPs were lifted to GRCh38 using UCSC liftover tool liftMap.py. For the truth set, variants with a genotype quality threshold of 30 was used and minor allele frequency of at least 5% in Hapmap v3 were used. Familial relationships within the WGS truth set, and between the truth set and the genotype data used for linkage analysis, were checked by inferred identity-by-descent analysis using plink v1.9. All relationships were as expected.

Effect alleles were set to the effect alleles reported in gnomAD release 2.0.1 (gnomad.genomes.r2.0.1.sites.GRCh38.noVEP.vcf.gz). Using snpSift, the following annotations were added: gnomAD non-Finnish European allele frequencies, variant information from the database for non-synonymous function prediction (dbNSFP), allele frequencies from the genome of the Netherlands (GoNL) database,^8^ and allele frequencies of controls from a large cohort of Dutch genomes (Project MinE).^9^

## TaqMan assay design and optimization

TaqMan assays specific for variants of interest were ordered from Thermo Fisher Scientific (Waltham, MA, USA). The assay for *TBC1D2* p.Thr754Lys was not readily available and therefore designed using the Thermo Fisher TaqMan assay design tool.

DNA samples with known genotypes obtained from WGS (heterozygous and homozygous ancestral) were used as positive controls. The amount of template DNA was optimized using a range of 12.5 – 50 ng per reaction to obtain the best cluster separation. Reactions were done in a 384-well plate containing 1μl of template DNA in the optimized concentration, 2.5μl of 2X TaqPath ProAmp Master Mix (Applied Biosystems), 0.125μl of 40X TaqMan SNP assay, and Milli-Q water in a total volume of 5 μl per well.

## Rare variant burden analysis

Exome sequencing genotype calls from 200,632 participants of the UK Biobank were used or rare variant burden analysis.^10^ Participants who withdrew consent for data use were excluded from the analysis. Intracranial aneurysm status or incidence of aneurysmal subarachnoid haemorrhage was assessed by ICD-10 codes I67.1 and I60, respectively, of hospital admittance, or cause of death. Participants with a connective tissue disease predisposing to intracranial aneurysms, being Ehlers-Danlos disease (Q79.6), Marfan’s syndrome (Q87.4), or polycystic kidney disease (Q61.1, Q61.2, Q61.3), were excluded.

Gene regions were obtained for genome reference GRCh38 from Ensembl version 104.^11^ Variants on coding regions of the genes *TBC1D2*, *ZNF782*, *CCDC180*, *NCBP1*, *FMNL2*, and *SYCP1*, with a genotyping rate of 98% or higher were retained. Predicted functional impact was annotated using SNPeff.^7^ Variants were binned into impact categories high, moderate + high, and low + moderate + high, for two categories of minor alleles: below 0.5%, and below 1% (also including variants below 0.5%). Number of minor alleles per gene in each of the respective variant categories was counted and used as independent variable in a two-tailed Firth logistic regression in R. The outcome was IA status, and sex, age, and the first ten genetic principal components were used as covariates.

## Variant level association analysis in the UK Biobank

To identify additional potentially pathogenic variants of the genes *TBC1D2*, *ZNF782*, *CCDC180*, *NCBP1*, *FMNL2*, and *SYCP1*, variants predicted to have a high functional impact by SNPEff,^7^ with a minor allele frequency below 1%, were selected. This analysis was done in the same cohort as described above for the burden analysis. Association between the variants and IA status was assessed using PLINK v2.0 (ref. ^12^) two-tailed Firth logistic regression with sex, age, and the first ten genetic principal components as covariates.

# Supplementary Figures


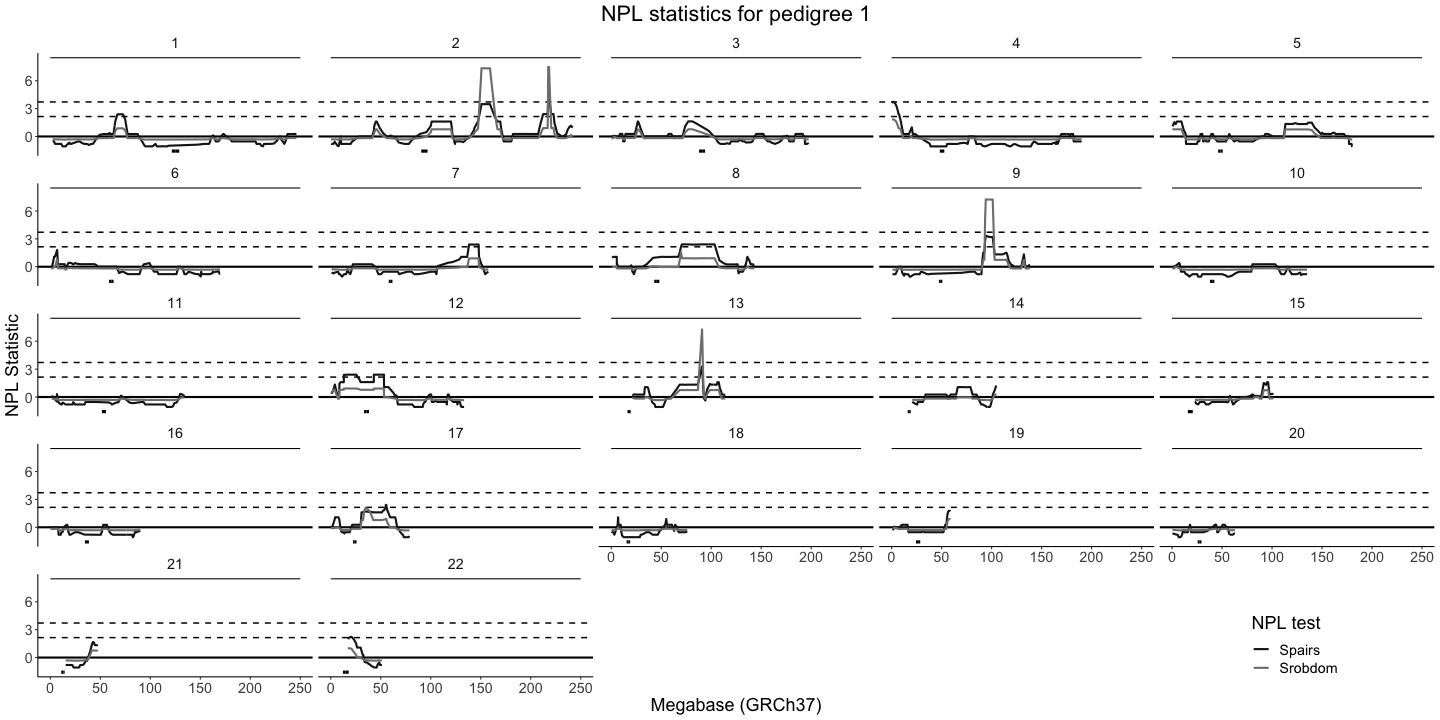


**Supplementary Figure 1. Non-parametric linkage (NPL) analysis of pedigree 1**. NPL statistics Spairs and Srobdom are shown on the vertical axis, with genomic position (×1,000,000 base pairs) on the horizontal axis. Each panel is a chromosome. Horizontal dotted lines correspond to suggestive and strong evidence for linkage (NPL = 2.15 and 3.90, respectively). Locations of centromeres are shown by thick black line segments near the horizontal axes.


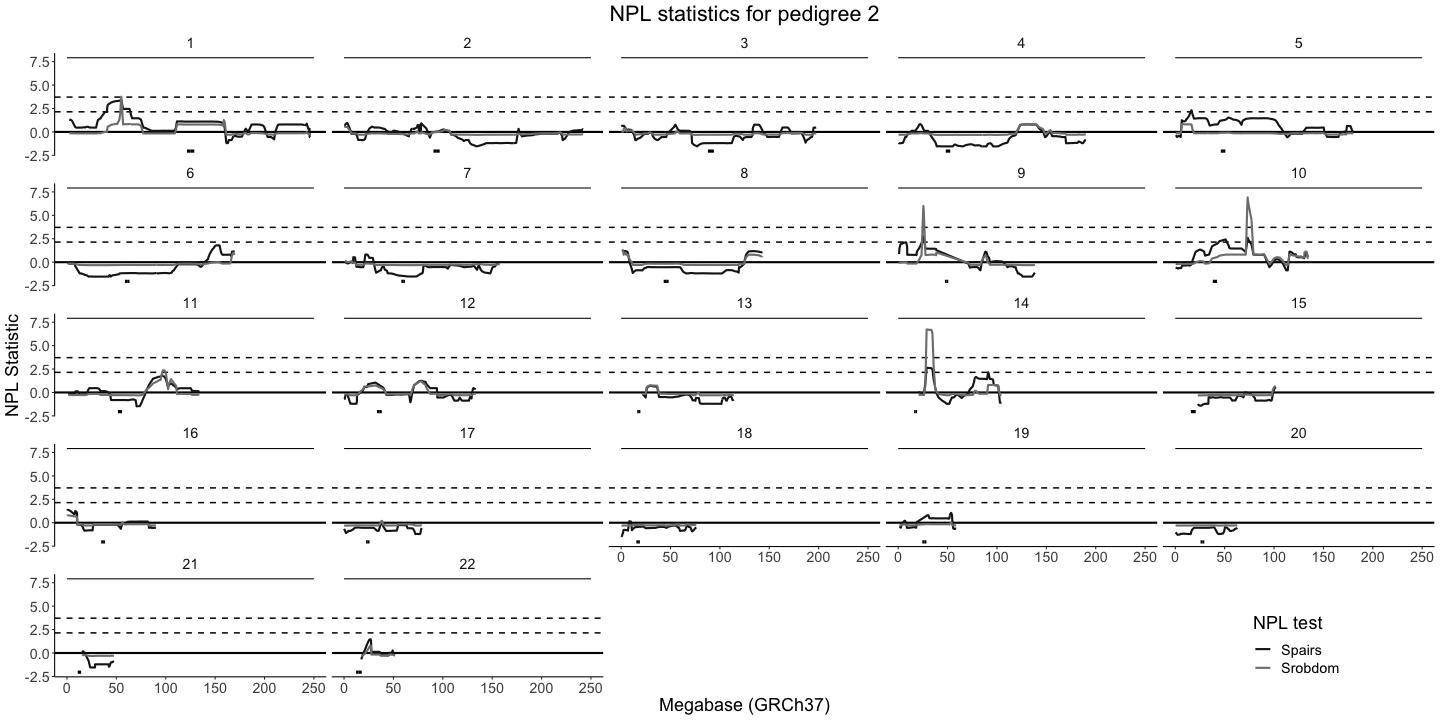


**Supplementary Figure 2. Non-parametric linkage (NPL) analysis of pedigree 2**. NPL statistics Spairs and Srobdom are shown on the vertical axis, with genomic position (×1,000,000 base pairs) on the horizontal axis. Each panel is a chromosome. Horizontal dotted lines correspond to suggestive and strong evidence for linkage (NPL = 2.15 and 3.90, respectively). Locations of centromeres are shown by thick black line segments near the horizontal axes.


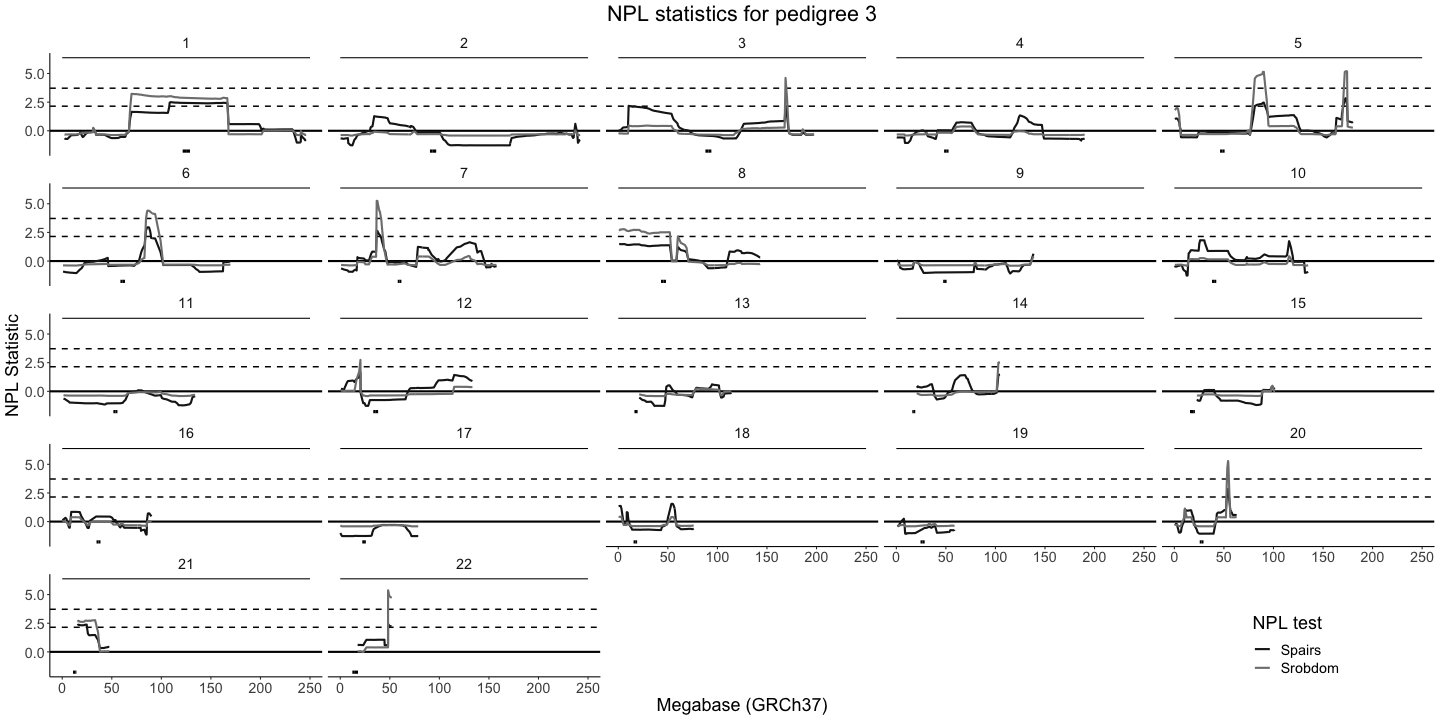


**Supplementary Figure 3. Non-parametric linkage (NPL) analysis of pedigree 3**. NPL statistics Spairs and Srobdom are shown on the vertical axis, with genomic position (×1,000,000 base pairs) on the horizontal axis. Each panel is a chromosome. Horizontal dotted lines correspond to suggestive and strong evidence for linkage (NPL = 2.15 and 3.90, respectively). Locations of centromeres are shown by thick black line segments near the horizontal axes.

**
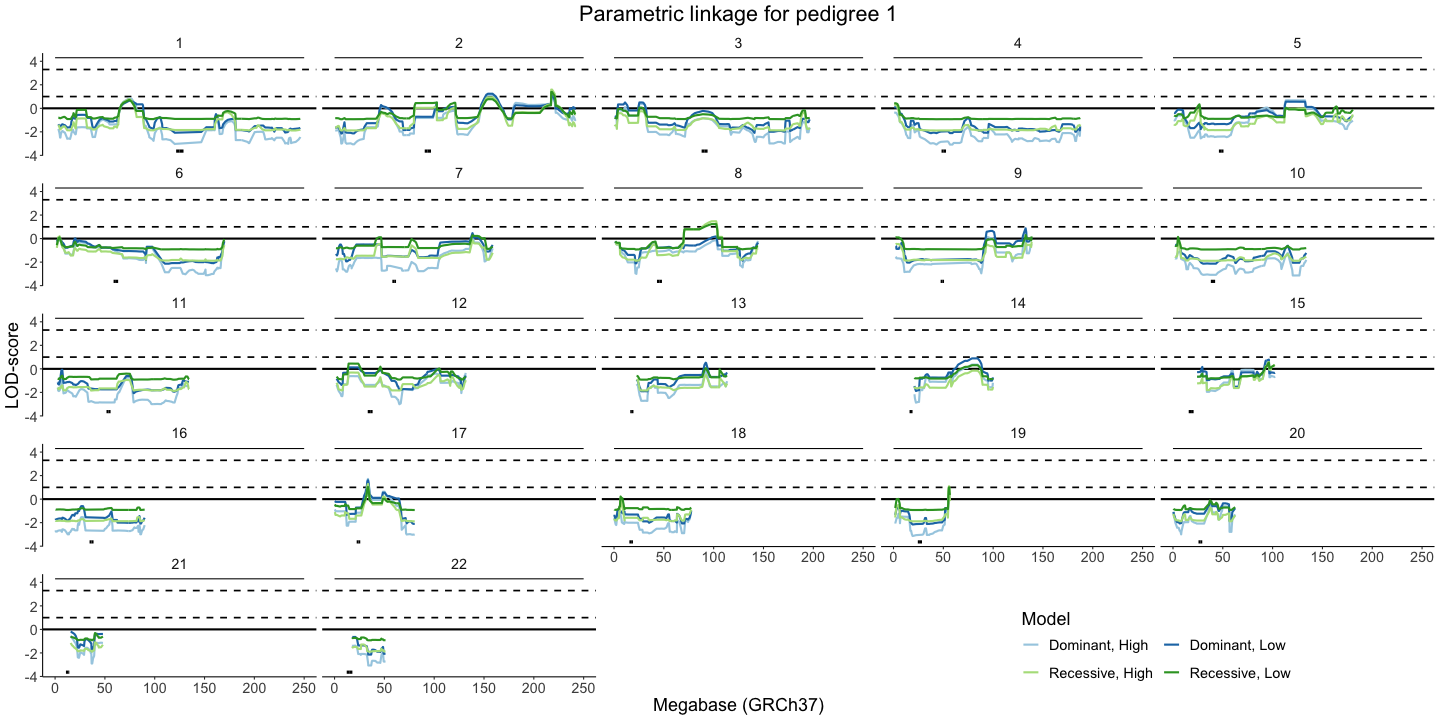
Supplementary Figure 4. Parametric linkage analysis of pedigree 1**. Logarithm-of-odds (LOD) scores are shown on the vertical axis for four penetrance models (dominant and recessive with low or high penetrance). Genomic position (×1,000,000 base pairs) on the horizontal axis. Each panel is a chromosome. Horizontal dotted lines correspond to suggestive and strong evidence for linkage (LOD = 1 and 3.3, respectively). Locations of centromeres are shown by thick black line segments near the horizontal axes.


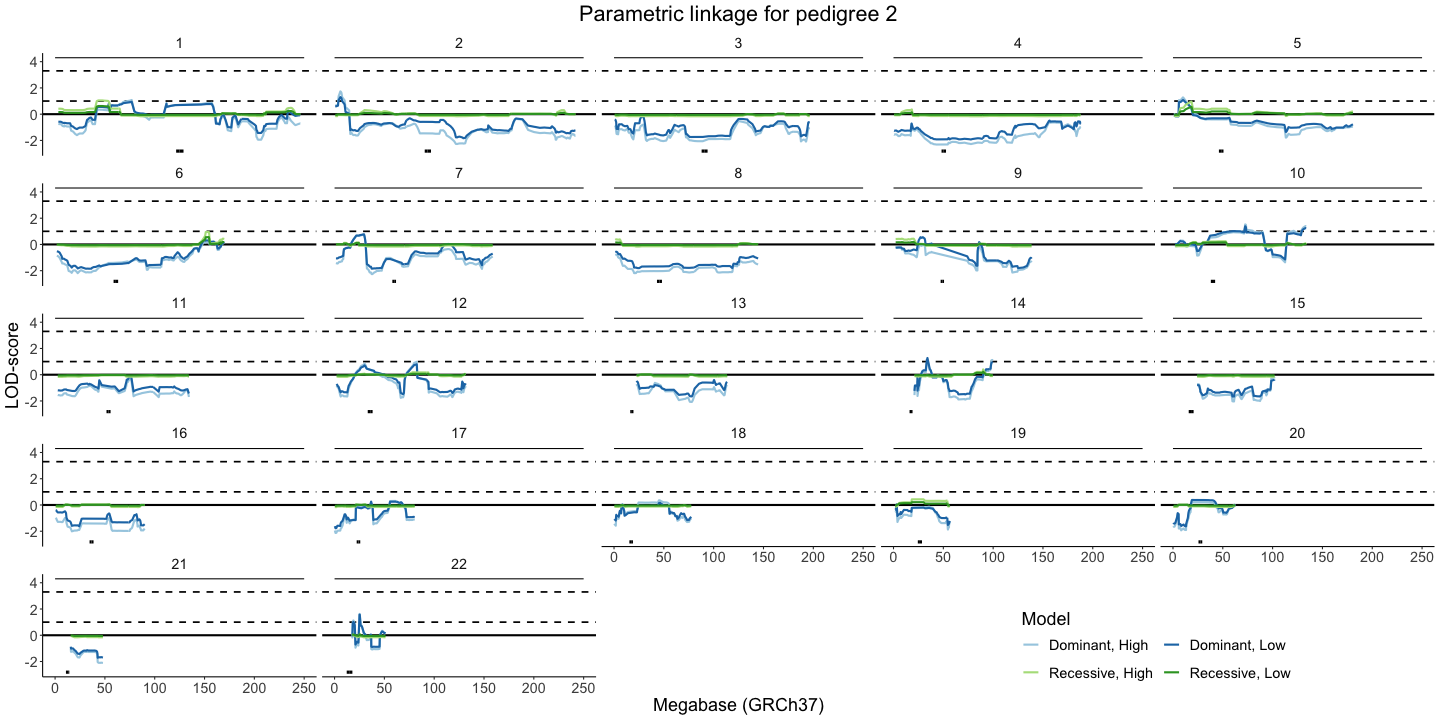


**Supplementary Figure 5. Parametric linkage analysis of pedigree 2**. Logarithm-of-odds (LOD) scores are shown on the vertical axis for four penetrance models (dominant and recessive with low or high penetrance). Genomic position (×1,000,000 base pairs) on the horizontal axis. Each panel is a chromosome. Horizontal dotted lines correspond to suggestive and strong evidence for linkage (LOD = 1 and 3.3, respectively). Locations of centromeres are shown by thick black line segments near the horizontal axes.


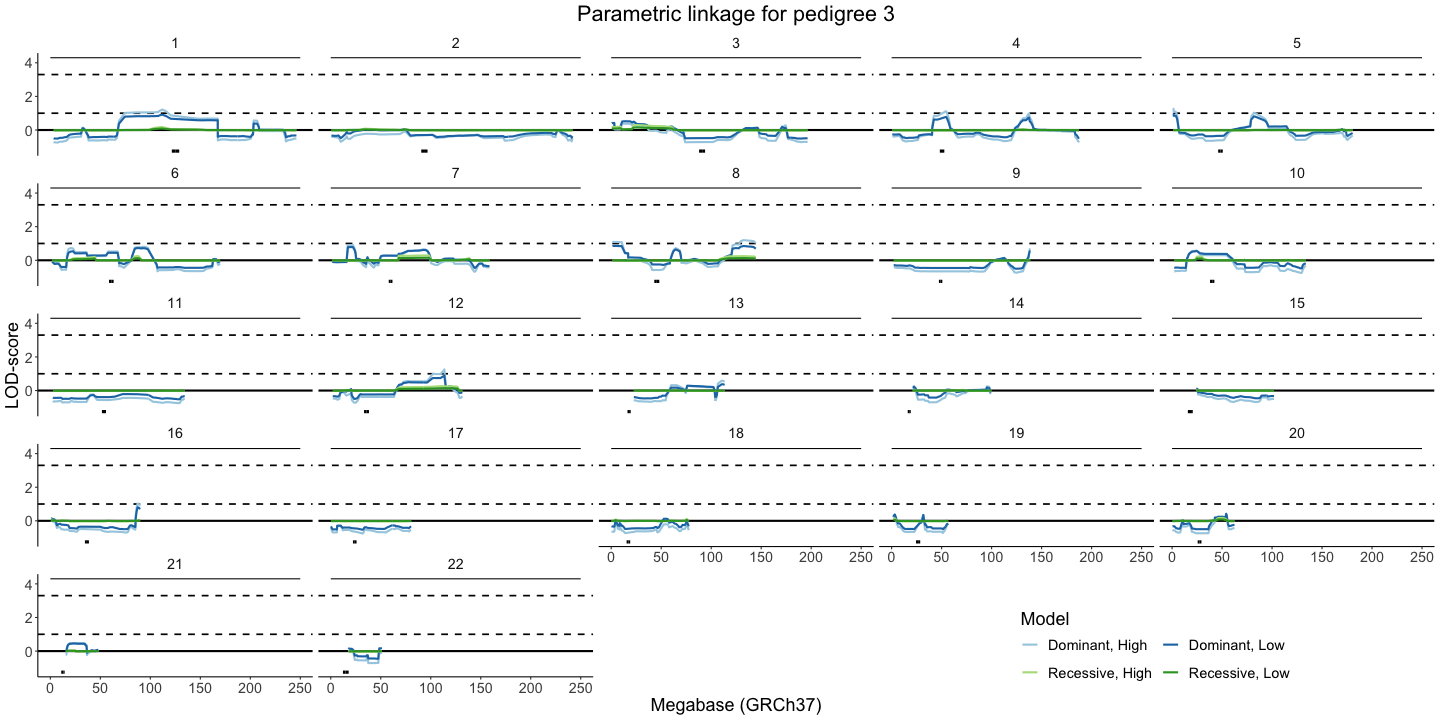


**Supplementary Figure 6. Parametric linkage analysis of pedigree 3**. Logarithm-of-odds (LOD) scores are shown on the vertical axis for four penetrance models (dominant and recessive with low or high penetrance). Genomic position (×1,000,000 base pairs) on the horizontal axis. Each panel is a chromosome. Horizontal dotted lines correspond to suggestive and strong evidence for linkage (LOD = 1 and 3.3, respectively). Locations of centromeres are shown by thick black line segments near the horizontal axes.

# Supplementary Tables

**Supplementary Table 1. Linkage loci used to supplement regions of linkage identified in the present study.**

| **Locus** | **Population** | **LOD-score** | **Reference** |
| --- | --- | --- | --- |
| 1p34.3–36.13 | North American | 4.2 | Nahed, et al. (2005)^13^ |
| 1p36.13-36.13 | Dutch | 3.18 | Ruigrok, et al. (2008)^14^ |
| 2p13.1-15 | Dutch | 3.55 | Roos, et al. (2004)^15^ |
| 4q32 | FIA | 2.5 | Foroud, et al. (2008)^16^ |
|  |  | 2.6 | Foroud, et al. (2009)^17^ |
| 5p15.2–14.3 | French-Canadian | 3.57 | Verlaan, et al. (2006)^18^ |
| 5q22-32 | Japanese | 2.24 | Onda, et al. (2001)^19^ |
| 7q11 | Utah | 2.34 | Farnham, et al. (2004)^20^ |
|  | Japanese | 3.22 | Onda, et al. (2001)^19^ |
| 8p22.2 | Korean | 3.61 | Kim, et al. (2011)^21^ |
| 11q24-25 | North American | 4.3 | Ozturk, et al. (2006)^22^ |
| 12p12.3 | FIA | 3.1 | Foroud, et al. (2009)^17^ |
| 13q14-21 | French-Canadian | 4.56 | Santiago-Sim, et al. (2009)^23^ |
| 14q22 | Japanese | 2.31 | Onda, et al. (2001)^19^ |
| 14q23-31 | North American | 3.0 | Ozturk, et al. (2006) ^22^ |
| 17cen | Japanese | 3.0 | Yamada, et al. (2004)^24^ |
| 19q13.3 | Finnish | 2.6 | Olson, et al. (2002)^25^ |
|  |  | 3.16 | van der Voet, et al. (2004)^26^ |
|  | Japanese | 4.1 | Mineharu, et al. (2007)^27^ |
|  |  | 2.15 | Yamada, et al. (2004)^24^ |
| Xp22.2-22.32 | Dutch | 4.54 | Ruigrok, et al. (2008) ^14^ |
| Xp22 | Finnish | 2.08 | Olson, et al. (2002)^25^ |
|  | Japanese | 2.16 | Yamada, et al. (2004)^24^ |

LOD: logarithm-of-odds. FIA: familial intracranial aneurysm study. Table adapted and supplemented from Zhou, et al. (2018).^28^

**Supplementary Table 2. Primers used for PCR amplification and Sanger sequencing.**

| **Variant** | **Chr** | **Position** | **Gene** | **Variant region** | **Forward primer** | **Reverse primer** | **T_ann_ (°C)** |
| --- | --- | --- | --- | --- | --- | --- | --- |
| rs146221635 | 1 | 114857466 | SYCP1 | TGGTAATTCTGACTGTCACT[A/G]TCAGGAAGGACTAAAADACT | TTGAGCAGGTCAGTTAAGCA | AATGGGAAATCTTCTGACCTC | 62 |
| rs182064759 | 2 | 152647857 | FMNL2 | AGAAGCGTCAGGCGSCGCTT[T/G]GATGATCAGAACTTGCGTTC | CACTGGCACTCTCCCCTTTA | TGTATTCATGCAGGCCAGTC | 61 |
| rs74447004 | 2 | 165933044 | TTC21B | TGCTTCCACATTTTGGCTAT[C/T]TTGGAGCAGCAACCTGCAGG | TGCACACATGGGTGTGTATG | GGCAAGCAAACCAACTTTTC | 58 |
| rs140205258 | 6 | 88891815 | RNGTT | RTATTTATTACCCTTCCCAG[C/T]CACAGAATTGATGACACTTC | TGCATTGATTGTCTTGAACGA | AACACAACCAAAGTTAGGAGAGG | 59.5 |
| rs138582676 | 9 | 92616050 | IPPK | TGAAGGGACGACAGGCCTTT[G/C]ATCAGARCTGCAAGTAAAAA | CGATCTTGCCGTCCAGTTTA | GGGAGGGAGGGAAATGAAT | 61 |
| rs146058964 | 9 | 96819061 | ZNF782 | GYACTGGGAGGGTTGAATTA[C/T]RGTTGAAACTTTTTCCATAT | GGGTTTTGCCCTTATGTGAA | CAGGAGGGAAATCCTTCAGC | 61 |
| rs183800704 | 9 | 97350488 | CCDC180 | AGGTGTCCCTGCGCAGCTTC[C/T]GGCAGTACTTGGAGGAGAGT | ACACACTGCACCAGGAGTTG | AAACTCAGGTGGCCTGAAGA | 62.5 |
| rs143745791 | 9 | 97643352 | NCBP1 | CAAACAATTATAAYGAAGCB[G/A]TGTATTTGGTAAGTTWGTTT | GAAGCCATGATTCGTCAACTT | TTTGGGGGTATCGTCAAAAT | 61 |
| chr9_98203298 | 9 | 98203298 | TBC1D2 | CCCCACTTACCTGGGATGCY[G/T]TCAGCGTGTTRCAGTAGTAA | TCCCAACTTTCCTGAGAGTCA | ATCACTCCAGGCCTTCCCTA | 61 |
| rs35544077 | 9 | 99054627 | COL15A1 | TCCTGGCTCAAAGGGARAAA[A/G]AGGCGACCAGGGANCCCAGG | GAGGGCTTTGTTTGAGTGGA | GGCAAGATTGAGAACAAGAGC | 61 |
| rs140417636 | 9 | 95447208 | PTCH1 | GGCAGTGGACGCTGGGTTCC[G/A]AGGGTTGTGAGAACVGGCCC | CTTCCACCTTCGAATCCCTCCT | GTGAAACCCAAGGAGGGAAGTGTG | 67 |
| rs201102622 | 8 | 13099452 | DLC1 | TGCCKGTGCTGTCCADGTCG[C/A]TGGGTGTGGTTCGGTCGTTG | CAACCCCAGAATCCCTTCTT | TGAGGGAGATTCGGACTCAG | 58 |

Genome build is GRCh38. Variant region is the sequence surrounding the variants of interest according to Ensembl. T_ann_ is the optimized annealing temperature used in the PCR protocol. Chr: chromosome

**Supplementary Table 5. Variants identified by linkage analysis paired with genome sequencing.**

| **Pedigree** | **Chr** | **Position** | **ID** | **Effect allele** | **Other allele** | **MAF** | **Severity** | **CADD** | **Gene** |
| --- | --- | --- | --- | --- | --- | --- | --- | --- | --- |
| 3 | 1 | 114857466 | rs146221635 | G | A | 0.0043 | missense | 25.3 | *SYCP1* |
| 1 | 2 | 152647857 | rs182064759 | G | T | 0.0028 | missense | 16.62 | *FMNL2* |
| 1 | 2 | 165933044 | rs74447004 | T | C | 0.0069 | missense | 23.5 | *TTC21B* |
| 1 | 8 | 13099452 | rs201102622 | A | C | 1.50×10^-5^ | missense | 28.9 | *DLC1* |
| 1 | 9 | 92616050 | rs138582676 | C | G | 0.0033 | missense | 10.71 | *IPPK* |
| 1 | 9 | 95447208 | rs140417636 | A | G | 0.0014 | missense | 32 | *PTCH1* |
| 1 | 9 | 96819061 | rs146058964 | T | C | 0.0071 | missense | 6.721 | *ZNF782* |
| 1 | 9 | 97350488 | rs183800704 | T | C | 0.0018 | missense | 34 | *CCDC180* |
| 1 | 9 | 97643352 | rs143745791 | A | G | 0.0022 | structural interaction | 23.1 | *NCBP1* |
| 1 | 9 | 98203298 | - | T | G | - | missense | 18.14 | *TBC1D2* |
| 2 | 9 | 99054627 | rs35544077 | G | A | 0.0095 | missense | 23.8 | *COL15A1* |

Variants located on regions identified by linkage analysis in one of the three studied families, with all affected persons being heterozygous, were selected for genome sequencing in one of the families are shown. Chr: chromosome. Positions shown are on GRCh38. MAF: minor allele frequency in non-Finnish European persons included in the Exome Aggregation Consortium (ExAC). Severity is annotated by SnpEff. CADD: combined annotation dependent depletion score.

**Supplementary Table 6. Rare variant burden analysis of the six genes with variants segregating with IA status.**

| **Gene** | **MAF** | **Impact** | **OR (95% CI)** | **P** |
| --- | --- | --- | --- | --- |
| TBC1D2 | < 0.005 | High | 1.53 (0.03 - 5.08) | 0.69 |
| TBC1D2 | < 0.005 | Moderate + high | 0.97 (0.55 - 1.53) | 0.92 |
| TBC1D2 | < 0.005 | Low + moderate + high | 0.92 (0.58 - 1.36) | 0.71 |
| TBC1D2 | < 0.01 | High | 1.53 (0.03 - 5.08) | 0.69 |
| TBC1D2 | < 0.01 | Moderate + high | 1.01 (0.63 - 1.49) | 0.97 |
| TBC1D2 | < 0.01 | Low + moderate + high | 0.99 (0.73 - 1.29) | 0.94 |
| ZNF782 | < 0.005 | High | 0.65 (0.01 - 4.41) | 0.74 |
| ZNF782 | < 0.005 | Moderate + high | 1.25 (0.61 - 2.23) | 0.51 |
| ZNF782 | < 0.005 | Low + moderate + high | 1.26 (0.66 - 2.14) | 0.46 |
| ZNF782 | < 0.01 | High | 0.65 (0.01 - 4.41) | 0.74 |
| ZNF782 | < 0.01 | Moderate + high | 1.19 (0.71 - 1.85) | 0.49 |
| ZNF782 | < 0.01 | Low + moderate + high | 1.20 (0.74 - 1.82) | 0.44 |
| CCDC180 | < 0.005 | High | 0.84 (0.10 - 2.98) | 0.83 |
| CCDC180 | < 0.005 | Moderate + high | 0.87 (0.59 - 1.22) | 0.45 |
| CCDC180 | < 0.005 | Low + moderate + high | 1.01 (0.75 - 1.31) | 0.95 |
| CCDC180 | < 0.01 | High | 0.84 (0.10 - 2.98) | 0.83 |
| CCDC180 | < 0.01 | Moderate + high | 0.87 (0.64 - 1.15) | 0.33 |
| CCDC180 | < 0.01 | Low + moderate + high | 0.97 (0.75 - 1.23) | 0.80 |
| NCBP1 | < 0.005 | High | 0.80 (0.17 - 2.24) | 0.71 |
| NCBP1 | < 0.005 | Moderate + high | 0.87 (0.33 - 1.82) | 0.73 |
| NCBP1 | < 0.005 | Low + moderate + high | 0.92 (0.41 - 1.72) | 0.81 |
| NCBP1 | < 0.01 | High | 0.80 (0.17 - 2.24) | 0.71 |
| NCBP1 | < 0.01 | Moderate + high | 0.87 (0.33 - 1.82) | 0.73 |
| NCBP1 | < 0.01 | Low + moderate + high | 0.92 (0.41 - 1.72) | 0.81 |
| FMNL2 | < 0.005 | High | 3.47 (0.03 - 24.3) | 0.47 |
| FMNL2 | < 0.005 | Moderate + high | 0.81 (0.46 - 1.32) | 0.42 |
| FMNL2 | < 0.005 | Low + moderate + high | 0.96 (0.59 - 1.45) | 0.84 |
| FMNL2 | < 0.01 | High | 3.47 (0.03 - 24.3) | 0.47 |
| FMNL2 | < 0.01 | Moderate + high | 0.98 (0.65 - 1.42) | 0.93 |
| FMNL2 | < 0.01 | Low + moderate + high | 1.07 (0.73 - 1.50) | 0.72 |
| SYCP1 | < 0.005 | High | 2.53 (0.02 - 17.6) | 0.57 |
| SYCP1 | < 0.005 | Moderate + high | 0.59 (0.22 - 1.24) | 0.18 |
| SYCP1 | < 0.005 | Low + moderate + high | 0.69 (0.35 - 1.20) | 0.20 |
| SYCP1 | < 0.01 | High | 2.53 (0.02 - 17.6) | 0.57 |
| SYCP1 | < 0.01 | Moderate + high | 0.73 (0.37 - 1.26) | 0.27 |
| SYCP1 | < 0.01 | Low + moderate + high | 0.84 (0.53 - 1.25) | 0.42 |

MAF: minor allele frequency threshold. OR: odd ratio. CI: Confidence interval.

**Supplementary Table 8. Expression of IA-associated genes in aneurysm and cerebral artery tissue.**

| **Subset** | ***SYCP1*** | ***FMNL2*** | ***TBC1D2*** | ***ZNF782*** | ***CCDC180*** | ***NCBP1*** |
| --- | --- | --- | --- | --- | --- | --- |
| All | 0 [0, 0, 0, 0.11] | 11.1 [1.18, 8.07, 15.3, 38.5] | 10.3 [1.55, 5.23, 14.8, 61.9] | 0.62 [0.05, 0.44, 0.88, 2.66] | 0.24 [0, 0.13, 0.37, 1.60] | 4.74 [2.35, 4.03, 5.45, 8.54] |
| Aneurysms | 0 [0, 0, 0, 0.11] | 11.1 [1.18, 8.28, 14.3, 28.5] | 13.2 [2.31, 8.48, 18.1, 61.9] | 0.55 [ 0.07, 0.40, 0.77, 1.29] | 0.21 [0, 0.09, 0.31, 0.63] | 4.86 [2.35, 4.05, 5.50, 8.19] |
| Control cerebral artery | 0 [0, 0, 0, 0.06] | 11.1 [1.77, 7.84, 18.5, 38.5] | 6.80 [1.55, 3.78, 11.4, 28.0] | 0.81 [0.05, 0.53, 1.01, 2.66] | 0.29 [0.04, 0.19, 0.40, 1.60] | 4.54 [2.56, 4.00, 5.32, 8.54] |
| Ruptured aneurysm | 0 [0, 0, 0, 0.05] | 12.6 [1.18, 8.30, 15.0, 22.0] | 14.7 [2.32, 8.95, 24.8, 61.9] | 0.52 [0.07, 0.30, 0.64, 1.18] | 0.14 [0, 0.07, 0.21, 0.57] | 4.84 [2.35, 3.94, 5.71, 8.19] |
| Unruptured aneurysm | 0 [0, 0, 0, 0.11] | 9.75 [3.42, 8.19, 12.5, 28.5] | 11.7 [3.66, 8.24, 14.5, 18.8] | 0.67 [0.31, 0.46, 0.84, 1.29] | 0.29 [0, 0.23, 0.44, 0.63] | 4.89 [3.05, 4.30, 5.46, 6.20] |

Expression in reads per kilobase per million reads. Values are: median [min, Q1, Q3, max]. All aneurysms N=56, control cerebral artery N=51, ruptured aneurysm N=33, unruptured aneurysm N=23

# References

1 Cheung, C. Y., Thompson, E. A. & Wijsman, E. M. Detection of Mendelian consistent genotyping errors in pedigrees. *Genet Epidemiol* **38**, 291-299, doi:10.1002/gepi.21806 (2014).

2 Tong, L. & Thompson, E. Multilocus lod scores in large pedigrees: combination of exact and approximate calculations. *Hum Hered* **65**, 142-153, doi:10.1159/000109731 (2008).

3 [*https://www.bioinformatics.babraham.ac.uk/projects/fastqc/*](https://www.bioinformatics.babraham.ac.uk/projects/fastqc/).

4 McKenna, A. *et al.* The Genome Analysis Toolkit: a MapReduce framework for analyzing next-generation DNA sequencing data. *Genome Res* **20**, 1297-1303, doi:10.1101/gr.107524.110 (2010).

5 [*https://gatk.broadinstitute.org/hc/en-us/articles/360035535932*](https://gatk.broadinstitute.org/hc/en-us/articles/360035535932).

6 <https://broadinstitute.github.io/picard/>.

7 Cingolani, P. *et al.* A program for annotating and predicting the effects of single nucleotide polymorphisms, SnpEff: SNPs in the genome of Drosophila melanogaster strain w1118; iso-2; iso-3. *Fly (Austin)* **6**, 80-92, doi:10.4161/fly.19695 (2012).

8 van Leeuwen, E. M. *et al.* Genome of The Netherlands population-specific imputations identify an ABCA6 variant associated with cholesterol levels. *Nat Commun* **6**, 6065, doi:10.1038/ncomms7065 (2015).

9 van der Spek, R. A. A. *et al.* The project MinE databrowser: bringing large-scale whole-genome sequencing in ALS to researchers and the public. *Amyotroph Lateral Scler Frontotemporal Degener* **20**, 432-440, doi:10.1080/21678421.2019.1606244 (2019).

10 Szustakowski, J. D. *et al.* Advancing Human Genetics Research and Drug Discovery through Exome Sequencing of the UK Biobank. *MedRxiv*, doi:<https://doi.org/10.1101/2020.11.02.20222232> (2020).

11 Howe, K. L. *et al.* Ensembl 2021. *Nucleic Acids Res* **49**, D884-D891, doi:10.1093/nar/gkaa942 (2021).

12 Chang, C. C. *et al.* Second-generation PLINK: rising to the challenge of larger and richer datasets. *Gigascience* **4**, 7, doi:10.1186/s13742-015-0047-8 (2015).

13 Nahed, B. V. *et al.* Mapping a Mendelian form of intracranial aneurysm to 1p34.3-p36.13. *Am J Hum Genet* **76**, 172-179, doi:10.1086/426953 (2005).

14 Ruigrok, Y. M. *et al.* Genomewide linkage in a large Dutch family with intracranial aneurysms: replication of 2 loci for intracranial aneurysms to chromosome 1p36.11-p36.13 and Xp22.2-p22.32. *Stroke* **39**, 1096-1102, doi:10.1161/STROKEAHA.107.495168 (2008).

15 Roos, Y. B. *et al.* Genome-wide linkage in a large Dutch consanguineous family maps a locus for intracranial aneurysms to chromosome 2p13. *Stroke* **35**, 2276-2281, doi:10.1161/01.STR.0000141415.28155.46 (2004).

16 Foroud, T. *et al.* Genome screen to detect linkage to intracranial aneurysm susceptibility genes: the Familial Intracranial Aneurysm (FIA) study. *Stroke* **39**, 1434-1440, doi:10.1161/STROKEAHA.107.502930 (2008).

17 Foroud, T. *et al.* Genome screen in familial intracranial aneurysm. *BMC Med Genet* **10**, 3, doi:10.1186/1471-2350-10-3 (2009).

18 Verlaan, D. J. *et al.* A new locus for autosomal dominant intracranial aneurysm, ANIB4, maps to chromosome 5p15.2-14.3. *J Med Genet* **43**, e31, doi:10.1136/jmg.2005.033209 (2006).

19 Onda, H. *et al.* Genomewide-linkage and haplotype-association studies map intracranial aneurysm to chromosome 7q11. *Am J Hum Genet* **69**, 804-819, doi:10.1086/323614 (2001).

20 Farnham, J. M. *et al.* Confirmation of chromosome 7q11 locus for predisposition to intracranial aneurysm. *Hum Genet* **114**, 250-255, doi:10.1007/s00439-003-1044-z (2004).

21 Kim, C. J. *et al.* Identification of an autosomal dominant locus for intracranial aneurysm through a model-based family collection in a geographically limited area. *J Hum Genet* **56**, 464-466, doi:10.1038/jhg.2011.27 (2011).

22 Ozturk, A. K. *et al.* Molecular genetic analysis of two large kindreds with intracranial aneurysms demonstrates linkage to 11q24-25 and 14q23-31. *Stroke* **37**, 1021-1027, doi:10.1161/01.STR.0000206153.92675.b9 (2006).

23 Santiago-Sim, T. *et al.* Genomewide linkage in a large Caucasian family maps a new locus for intracranial aneurysms to chromosome 13q. *Stroke* **40**, S57-60, doi:10.1161/STROKEAHA.108.534396 (2009).

24 Yamada, S. *et al.* Genome-wide scan for Japanese familial intracranial aneurysms: linkage to several chromosomal regions. *Circulation* **110**, 3727-3733, doi:10.1161/01.CIR.0000143077.23367.18 (2004).

25 Olson, J. M. *et al.* Search for intracranial aneurysm susceptibility gene(s) using Finnish families. *BMC Med Genet* **3**, 7, doi:10.1186/1471-2350-3-7 (2002).

26 van der Voet, M. *et al.* Intracranial aneurysms in Finnish families: confirmation of linkage and refinement of the interval to chromosome 19q13.3. *Am J Hum Genet* **74**, 564-571, doi:10.1086/382285 (2004).

27 Mineharu, Y. *et al.* Model-based linkage analyses confirm chromosome 19q13.3 as a susceptibility locus for intracranial aneurysm. *Stroke* **38**, 1174-1178, doi:10.1161/01.STR.0000259657.73682.03 (2007).

28 Zhou, S., Dion, P. A. & Rouleau, G. A. Genetics of Intracranial Aneurysms. *Stroke* **49**, 780-787, doi:10.1161/STROKEAHA.117.018152 (2018).
